# Phylogenetic inference of pneumococcal transmission from cross-sectional data, a pilot study

**DOI:** 10.12688/wellcomeopenres.19219.1

**Published:** 2023-10-06

**Authors:** Jada Hackman, Carmen Sheppard, Jody Phelan, William Jones-Warner, Ben Sobkowiak, Sonal Shah, David Litt, Norman K. Fry, Michiko Toizumi, Lay-Myint Yoshida, Martin Hibberd, Elizabeth Miller, Stefan Flasche, Stéphane Hué

**Affiliations:** 1 Faculty of Epidemiology and Population Health, London School of Hygiene and Tropical Medicine, London, UK; 2 School of Tropical Medicine and Global Health, Nagasaki University, Nagasaki, Japan; 3 Vaccine Preventable Bacteria Section, UK Health Security Agency, London, UK; 4 Faculty of Infectious and Tropical Diseases, London School of Hygiene and Tropical Medicine, London, UK; 5 Immunisation & Countermeasures Division, UK Health Security Agency, London, UK; 6 Department of Paediatric Infectious Diseases, Nagasaki University, Nagasaki, Japan

**Keywords:** within-host diversity, phylogenetic, transmission direction, Streptococcus pneumoniae, pneumococcus

## Abstract

**Background:** Inference on pneumococcal transmission has mostly relied on longitudinal studies which are costly and resource intensive. Therefore, we conducted a pilot study to test the ability to infer who infected whom from cross-sectional pneumococcal sequences using phylogenetic inference.

**Methods:** Five suspected transmission pairs, for which there was epidemiological evidence of who infected whom, were selected from a household study. For each pair, *Streptococcus pneumoniae* full genomes were sequenced from nasopharyngeal swabs collected on the same day. The within-host genetic diversity of the pneumococcal population was used to infer the transmission direction and then cross-validated with the direction suggested by the epidemiological records.

**Results:** The pneumococcal genomes clustered into the five households from which the samples were taken. The proportion of concordantly inferred transmission direction generally increased with increasing minimum genome fragment size and single nucleotide polymorphisms. We observed a larger proportion of unique polymorphic sites in the source bacterial population compared to that of the recipient in four of the five pairs, as expected in the case of a transmission bottleneck. The only pair that did not exhibit this effect was also the pair that had consistent discordant transmission direction compared to the epidemiological records suggesting potential misdirection as a result of false-negative sampling.

**Conclusions:** This pilot provided support for further studies to test if the direction of pneumococcal transmission can be reliably inferred from cross-sectional samples if sequenced with sufficient depth and fragment length.

## Introduction

Pneumococcal disease is a major contributor to global mortality amongst children less than five years old ( O’Brien *et al.*, 2009; Wahl *et al.*, 2018). The main route of *Streptococcus pneumoniae* ( *Sp*) transmission is through close physical interpersonal contact and exposure to contaminated respiratory secretion ( le Polain de Waroux *et al.*, 2018; Neal *et al.*, 2019; van der Poll & Opal, 2009). Children are the main reservoir for infection and transmission ( Flasche *et al.*, 2020; Qian *et al.*, 2022; Weinberger *et al.*, 2019; Zivich *et al.*, 2018). Reduction of vaccine-type carriage via pneumococcal conjugate vaccines enhances direct vaccine impact beyond the vaccinated children by mitigating onward spread ( Grijalva *et al.*, 2007; O’Brien & Dagan, 2003; Poolman *et al.*, 2013; Principi & Esposito, 2016). With a more in-depth understanding of pneumococcal transmission, vaccination strategies may be further improved, but classical epidemiological approaches to understanding transmission rely on time and resource-intensive longitudinal studies.

Phylogenetic inference is particularly well suited for the exploration of infectious disease dynamics at the between-host and within-host level and may allow inference of transmission even from more easily collected cross-sectional infection surveys, including those for pneumococcal carriage ( Gouliouris *et al.*, 2021; PANGEA Consortium and Rakai Health Sciences Program *et al.*, 2019; Xu *et al.*, 2020). The phylogenetic analysis of pathogen genomes sampled from an infected population in principle not only allows the identification of transmission partners or clusters but also, the direction of transmission (who infected whom) ( Rose *et al.*, 2020; Zhang *et al.*, 2021). These approaches have so far been mainly developed for and applied to study viral pathogens, particularly human immunodeficiency virus (HIV) and hepatitis C virus ( Hall *et al.*, 2019; Jacka *et al.*, 2014; Leitner, 2019; Rose *et al.*, 2020; Street *et al.*, 2020).

Phyloscanner is a phylogenetic algorithm that infers the direction of transmission from similarities in within-host pathogen diversity. Until the development of Phyloscanner, most of the available tools lacked sufficient sensitivity to infer the direction of transmission due to limited use of the within-host genetic signal ( Wymant *et al.*, 2018). Moreover, Phyloscanner has been validated in the context of HIV direction of transmission with high concordance with the epidemiological records ( Zhang *et al.*, 2021).

Bacteria’s large genome size, slow rates of evolution, and frequent horizontal gene transfer characteristics make the application of phylogenetic approaches more difficult for these organisms than for most viruses. The decrease in genetic diversity that accompanies the transmission bottleneck limits the amount of genetic information that is detectable even further ( Worby *et al.*, 2014). A weak transmission bottleneck is needed to detect an adequate amount of within-host genetic diversity in both source and recipient to assess transmission linkage and its direction ( Didelot *et al.*, 2016). Despite these inherent limitations, the methodology applied to viral infectious diseases could still be applicable to bacterial infectious diseases.

This pilot study explored within-host pneumococcal bacterial diversity from whole-genome next-generation sequencing (NGS) data. We tested and adapted currently available phylogenetic approaches to infer linked pneumococcal infections and their transmission direction from cross-sectional pneumococcal carriage data.

## Methods

### Ethical considerations

Ethical approval for the study was granted by LSHTM’s institutional ethics board on 26 September 2019 (reference number: 17642). The samples were not collected as a part of a clinical trial and records of the consent forms, signed over 20 years ago, have not been stored. However, the samples included in this study were anonymized and were received as cultures for processing and did not include any human material and therefore, not covered by the Human Tissue Act.

### Study design and study samples

This study cohort was from a prospective, longitudinal household study of pneumococcal colonisation conducted in the county of Hertfordshire, United Kingdom in 2001–2002. The original study is described in detail elsewhere ( Hussain *et al.*, 2005). In summary, preschool children and their household contacts were enrolled and followed up monthly for 10 consecutive months. At each visit, nasopharyngeal swabs were collected and any *S. pneumoniae* bacteria isolated by culture were serotyped using DNA microarray or the Quellung reaction to identify carriage type ( Southern *et al.*, 2018).

A total of 10 within-household putative source-recipient transmission events were included based on the following inclusion criteria which were also the epidemiological evidence supporting a transmission event and its direction: (i) the recipient is tested positive for carrying a single pneumococcal serotype, (ii) the potential source of infection is an individual within the same household who was carrying the same serotype in the month before the recipient was tested positive, and (iii) in the two visits prior to the carriage episode of the recipient, the remainder of the household were found to not carry pneumococci of the same serotype ( Table 1).

**Table 1.** Samples were selected for inference of the direction of transmission of *S. pneumoniae* and within-host diversity. 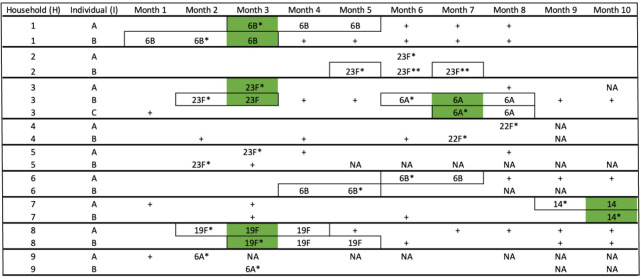

Green highlights paired same-visit samples used to infer the direction of transmission Box line highlights consecutive-visit samples used to estimate the within-host evolutionary rate * Paired subsequent-visit samples used for the sensitivity analysis ** Discordant serotyping between epidemiological data and genomic serotyping data for individual H2IB at month 6 *+* A positive nasal swab for pneumococci, but samples were not included in the analysis because they did not satisfy the epidemiological inclusion criteria Empty cell, a negative test for pneumococcal carriage “NA”, samples that were not obtained in the respective month

The epidemiological inclusion criteria aimed to maximise the probability of correctly identifying a transmission pair. In five instances, the source also carried pneumococci of the same serotype on the following visit resembling cross-sectional sampling of source and recipient. These five same-visit paired samples were used for the main direction of transmission analysis. We defined the sample ID in the following format: household (H), individual ID (I), and the month the swab was collected from (M); e.g. sample H1IAM1 was collected from household 1, individual A, from month 1 of the study.

The selected study samples were included in two different analyses:

(i) The main analysis tested the direction of transmission and included same-visit swabs of putative transmission pairs (N=10 individuals), simulating a cross-sectional carriage survey. Of the five pairs where same-visit samples were available, two pairs had a second same-serotype same-visit instance to assess within-host diversity ( Table 1). The same-visit samples were additionally used to estimate the proportion of unique single-nucleotide polymorphism (SNP) in the source-recipient pairs. Alongside this, the 10 pairs (N=20 individuals) where samples of source and recipient were taken from subsequent visits (one month apart) were used to also test the direction of transmission to assess the sensitivity of the method on more temporally distant samples. (ii) The second analysis was to estimate the within-host evolutionary rate from 10 individuals who had at least two consecutive swabs of the same serotype (N=25 sequences) ( Table 1).

### Isolate culturing and whole-genome sequencing

Isolates were grown overnight on Columbia agar with horse blood (Oxoid, cat. No. #PB0122). The isolates used were from stock cultures stored at -80°C in glycerol blood broth medium (nutrient broth No. 2 (Oxoid) containing 15% glycerol (Fisher Scientific) and 4.8% fresh sterile defibrinated horse blood (TCS Bioscience)) since 2001/2002. The stocks used were pneumococcal isolates obtained from the culture plates directly inoculated with the swab in the original study. Samples from the glycerol blood broths were partially thawed when plated and DNA was extracted from entire plate growths using QIAsymphony SP automated instrument (Qiagen) and QIAsymphony DSP DNA Mini Kit and the manufacturer’s recommend tissue extraction protocol for Gram negative bacteria, which included a 1-hour pre-incubation with proteinase K in ATL buffer and RNAse A treatment. DNA concentrations were measured using the Quant-iT dsDNA Broad-Range Assay Kit (Life Technologies, Paisley, UK) and GloMax R © 96 Microplate Luminometer (Promega, Southampton, UK) to test for a minimum concentration of 20 ng/uL ( Kapatai *et al.*, 2016).

Whole-genome sequencing was carried out on the Illumina MiSeq platform on the DNA extracts. Library preparation was done using QIAseq FX DNA Library Kit (96 – Cat no:180475) as per the manufacturer's protocol yielding a DNA fragment size of 300 bp, including adaptors. Sequencing was completed using the Illumina Miseq in conjunction with the MiSeq Reagent Kit v2 (300-cycles – Cat no: MS-102-2002). The sequencing was run in duplicates and were later merged. Adaptors were removed from the raw sequencing data using Trimmomatic v0.39, along with low-quality reads based on an average quality and sliding window approach ( Bolger *et al.*, 2014). Additional quality control of the reads was carried out with Kraken2 v2.0.9 and unmatched *S. pneumoniae* reads were filtered out from the downstream analysis ( Wood *et al.*, 2019).

### Genomic serotyping

Genomic serotyping of the isolates was carried out on the *S. pneumoniae* sequencing reads using SeroBA v1.0.1, a tool that predicts pneumococcal serotypes using a k-mer-based approach from raw fastq data ( Epping *et al.*, 2018). Then the reads were aligned to a reference genome strain KK0981 (serotype 3, GenBank accession number AP017971) with the Burrow-Wheeler Alignment (BWA-MEM) and SAMtools mpileup software ( Chiba *et al.*, 2017; Li *et al.*, 2009; Li, 2013). Variant calling format files (VCF) containing information on SNP were generated using Freebayes v1.3.2 ( Garrison & Marth, 2012). A consensus sequence of all polymorphic positions was generated for each of the isolates which were then included in the phylogenetic reconstruction to identify linkage.

### Multi-carriage detection

**Figure 1.**
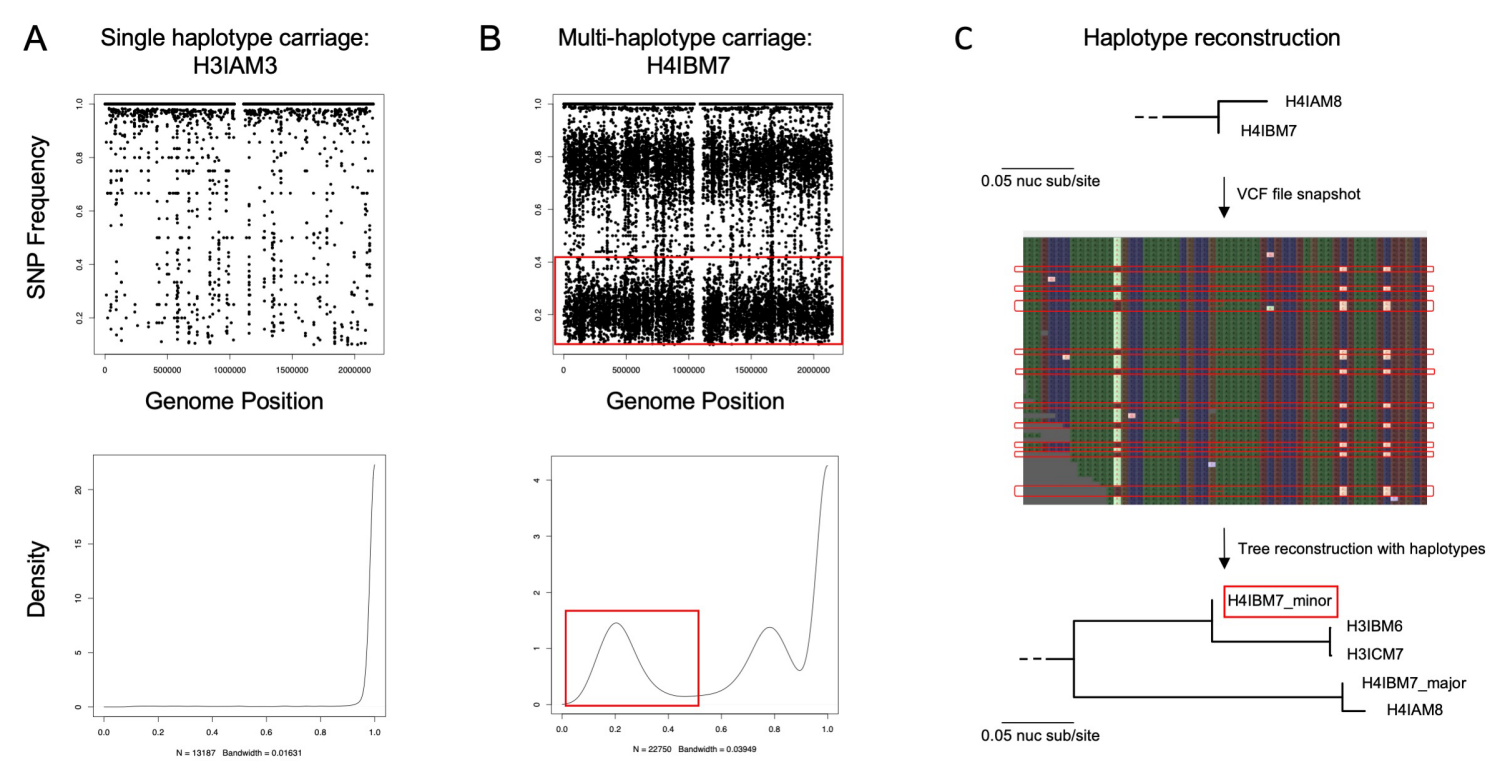
Haplotype reconstruction. ( **A**) This is an example where there is no evidence to support that the individual is infected with multiple haplotypes. A single point on the SNP frequency plot represents a single polymorphic site to the reference genome. SNP that occur at a frequency of 1.0 indicate the SNP is present in all of the sample’s reads while the density plot shows the density of the SNP frequencies. ( **B**) This is an example where there is evidence to support that the individual is infected with multiple haplotypes. The points on the SNP frequency plot reveal there are two populations with distinct clusters of polymorphic sites at 20% and 80% likewise in the density plot. The distribution occurring at 20% is designated as the minor strain and is highlighted in a red box throughout. ( **C**) Shows a snapshot of the phylogenetic consensus SNP tree with H4IBM7 (no haplotype isolation) and the linked isolate, H4IAM8. The snapshot of the variant calling format files highlights reads that correspond to the minor strain while the remainder corresponds to the major strain. The phylogenetic consensus SNP tree reconstruction after haplotype isolation reveals clustering of H4IBM7_major and H4IAM8 while H4IAM8_minor is more distantly related to H4IAM8.

We tested samples for multiple pneumococcal populations by assessing the distribution of SNP frequencies in all of the samples using LoFreq, a sensitive-variant calling tool ( Wilm *et al.*, 2012). The presence of more than one cluster or peak of SNP was considered as evidence for carriage of multiple-haplotypes, under the assumption that clusters of SNP are associated with common polymorphic sites within the reads ( Figure 1).

### Phylogenetic reconstruction of putative transmission pairs

The phylogenies of the sequenced bacterial genomes were reconstructed by maximum-likelihood inference using RAxML v2.0.2, under the General Time Reversible model of nucleotide substitutions and with 1,000 bootstrap replicates from the alignment of consensus single-nucleotide polymorphisms ( Stamatakis, 2014). Transmission pairs were identified from the consensus SNP tree topology as clusters of sequences (≤0.10 nuc sub/site) with branch support ≥90%.

### Inference of transmission direction

The most likely direction of transmission within a transmission pair was inferred using Phyloscanner v1.4.7 ( Wymant *et al.*, 2018). Each phylogeny inferred from Phyloscanner was classified as one of the following three relationships (i) single ancestry, where the subgraphs from the two populations form a paraphyletic (source) - monophyletic (recipient) relationship, (ii) equivocal, where the source and recipient subgraphs form dual monophyletic groups and thus the direction of infection is unclear, and (iii) complex ancestry, where the subgraphs form paraphyletic - paraphyletic groups and where the ancestral state is assigned to both the source and recipient depending on the subgraph ( Chiba *et al.*, 2017). The sub-trees, relationships identified with reads within a restricted sliding window, are then aggregated and the one that occurs the most often was considered to be the most likely scenario for the pair of individuals analysed. See Wymant *et al.* for more details on the methods implemented ( Wymant *et al.*, 2018).

**Figure 2.**
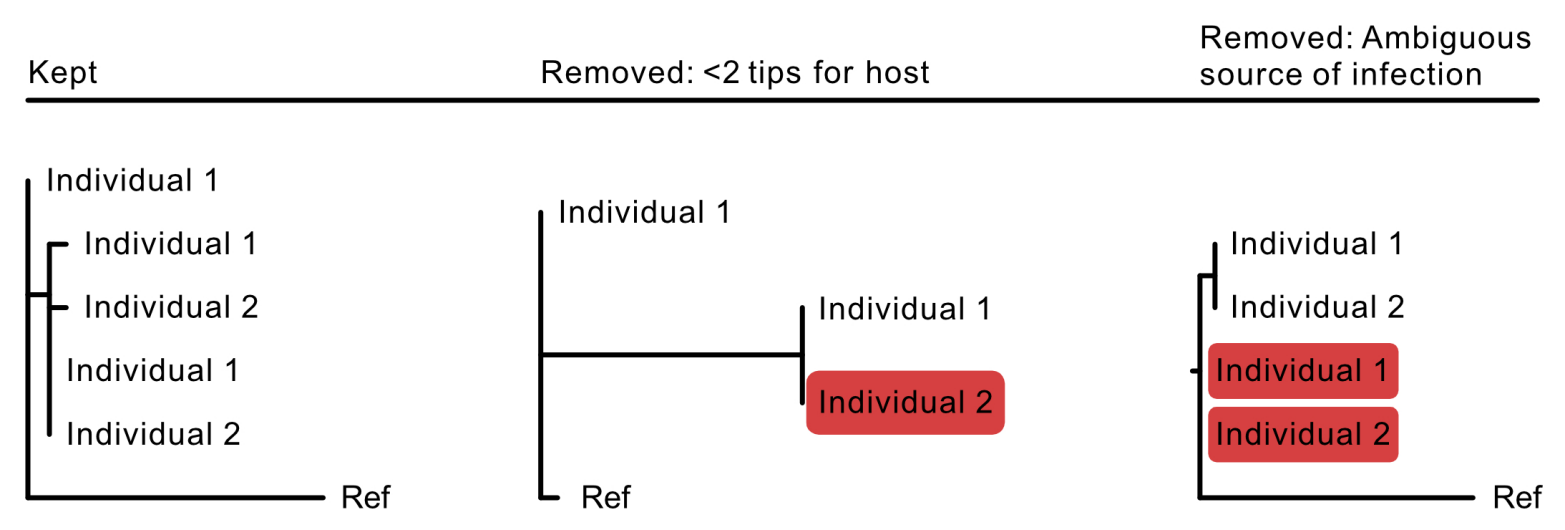
Two additional quality control steps were included in the direction of transmission analysis. **A**Shows a simplified sub-tree that would pass the quality control steps and would be included in the call for directionality where individual 1 is the source of the infection. ( **B**) Highlights the first step of the quality control which was to exclude sub-trees that were revealed to have only one tip from either individual (highlighted in red). ( **C**) Highlights the second step which is the excluded sub-trees that demonstrate both individuals being equally the source of the infection (highlighted in red).

Given the size of the pneumococcal genome analysed, approximately 2.1 million bp, and its low mutation rate, we restricted Phyloscanner to only process those windows that contained a predefined minimum number of SNP across the reads (1,3,5,7,9,11,13, or 15 SNP), to increase phylogenetic signal, and tested a range of window sizes. In addition, sub-trees (i) that had less than two tips from each host and (ii) where sequences from both hosts were equidistant from the reference sequence used as an outgroup were excluded to further enhance the accuracy of the inference ( Figure 2). This approach was used for the inference of transmission direction from both the same-visit and the subsequent-visit pairs.

As a sensitivity analysis to test the presence of bias in the inference in direction, the Phyloscanner analysis was carried out using reference strain ATCC700669 (serotype 23F, GenBank accession: NC_011900) as the mapping genome ( Croucher *et al.*, 2009). 

### Identifying unique SNP among source recipient pairs

The count and proportion of unique SNP detected in both members of a suspected transmission pair were estimated from the VCF files containing polymorphic sites mapped to the reference genome. The average percent of unique SNP in each individual was reported with standard deviation.

### Comparison of within-host diversities

*S. pneumoniae* within-host rate of nucleotide substitution, expressed as the number of nucleotide substitution/site/year, was estimated from the number of unique polymorphic sites accumulated between consecutive pneumococcal isolates from the same individual using the same methods as the proportion of unique SNP in recipient-source pairs.

## Results

### Streptococcus pneumoniae study samples

The bacterial populations analysed in this study are from a prospective longitudinal household pneumococcal colonisation study ( Hussain *et al.*, 2005). The previous study enrolled and followed 121 families in monthly intervals for 10 consecutive visits. The carriage prevalence was 52% for children 0–2 years old, 45% for 3–4 years, 21% for 5–17 years, and 8% for ≥18-year-old adults. A total of 10 transmission events across nine households met this study’s inclusion criteria where there is epidemiological evidence to support a transmission event and its direction.

Across the nine households, 37 samples were connected to suspected transmission events and were thus sequenced. Among those, five pairs containing the same serotypes were available and thus included in the main direction of transmission analysis (same-visit samples). Moreover, there were 10 pairs containing the same serotypes that were collected one month apart that were included in the sensitivity analysis (subsequent-visit samples). There were 10 individuals that had swabs with the same serotype across consecutive visits and thus included in the within-host evolutionary rate estimation. Of these 10 individuals, five had up to three consecutive swabs while the others had up to two ( Table 1).

### Whole-genome sequencing and sequence quality control

The isolates were cultured and whole plate scrapes were processed for whole-genome sequencing using Illumina MiSeq. The mean sequencing coverage of the genomes was 112 reads per position (standard deviation (SD), 31 reads), with the lowest mean coverage of 26 reads per position (samples H3IAM3 and H9IBM3) and the highest of 337 reads per position (sample H1IBM2). Overall, 85.6% (± 9.7) of the raw reads matched with *S. pneumoniae* genomic positions (range, 33.1% (H9IBM3) – 93.0% (H9IAM2)), and unmatched reads were filtered out for the downstream analysis ( Table 2).

**Table 2.** Sequencing quality of the whole-genome next-generation sequencing (NGS) reads for all 37 isolates included in the study.

ID	Mean Coverage	SD	Streptococcus pneumoniae read match (%)
H1IAM3	53	24	81.1
H1IAM4	130	47	84.2
H1IAM5	200	70	84.9
H1IBM1	186	66	86.7
H1IBM2	337	135	90.7
H1IBM3	189	66	85.0
H2IAM6	95	39	90.6
H2IBM5	71	30	87.6
H2IBM6	151	52	84.6
H2IBM7	141	56	80.0
H3IAM3	26	19	77.1
H3IBM2	91	37	92.9
H3IBM3	169	66	86.9
H3IBM6	76	29	90.7
H3IBM7	182	68	85.5
H3IBM8	166	61	83.9
H3ICM7	80	32	92.4
H3ICM8	168	62	85.8
H4IAM8	133	55	92.6
H4IBM7	67	25	91.0
H5IAM3	128	53	89.7
H5IBM2	90	37	90.4
H6IAM6	115	48	90.3
H6IAM7	50	19	88.6
H6IBM4	42	18	85.4
H6IBM5	110	41	85.4
H7IAM10	116	48	80.2
H7IAM9	66	28	89.2
H7IBM10	86	49	90.2
H8IAM2	59	27	91.8
H8IAM3	106	44	83.4
H8IAM4	103	43	83.3
H8IBM3	75	32	90.9
H8IBM4	103	43	82.6
H8IBM5	125	51	84.0
H9IAM2	39	17	92.9
H9IBM3	26	26	33.1

### Serotyping

**Figure 3.**
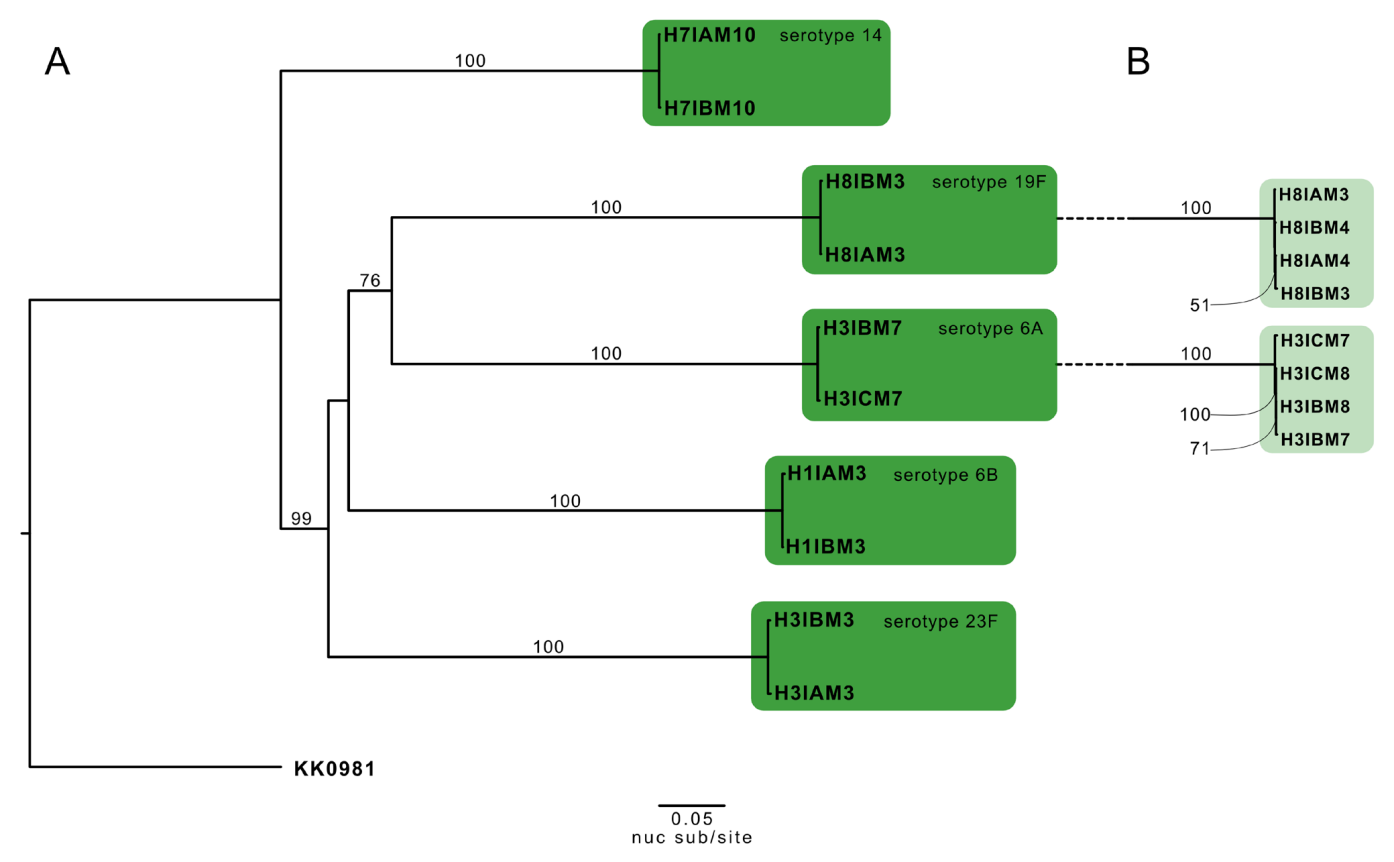
Maximum-likelihood phylogeny of the 10 *S. pneumoniae* genomes from five same-visit putative transmission pairs rooted to the reference genome, KK0981. ( **A**) The consensus single nucleotide polymorphism (SNP) tree was reconstructed from an alignment of polymorphic sites along the genomes (42,499 base pairs). Branch supports ≥50%, as determined by 1,000 bootstrap replicates, are denoted on the relevant branches. Branch length represents nucleotide substitutions per site (nuc sub/site), as denoted by the scaled bar. Clusters of two sequences supported by bootstrap score ≥90% were considered as putative transmission pairs and are highlighted by the dark green boxes. ( **B**) An additional same-visit transmission pair was included from household three (H3IBM8 and H3ICM8) and household eight (H8IAM4 & H8IBM4). The light green boxes highlight intermingling of transmission pairs with their respective within-host longitudinal swabs.

Of the 37 samples, 29 were previously serotyped using DNA microarray, while the remaining eight were serotyped using the Quellung reaction. For quality assurance, the isolates were then serotyped from the raw NGS reads using SeroBA genomic serotyping tool. The sequence-based serotype assignments were concordant with microarray serotyping, except for three of the 37 samples. Samples H9IAM2 and H9IBM3 were both originally identified as serotype 6A using the Quellung reaction but as 6C in the genomic serotyping. This was due to the reclassification of sub-lineages of serotype 6A to 6C subsequent to the original serotyping ( Park *et al.*, 2007). Furthermore, all three consecutive-visit samples from individual H2IB were classified as serotype 23F according to the microarray typing, however, sequence-based methods determined swab H2IBM6 as serotype 6B while H2IBM5 and H2IBM7 were concordant with the microarray data. Since we could not exclude the possibility that this discrepancy was the result of a sample mix-up, isolate H2IBM6 was excluded from the analysis but the subsequent-visit samples from H2IB were still included in the sensitivity analysis ( Figure 3A).

### Multiple-carriage’s role in phylogenetic tree reconstruction

Samples were tested for the presence of multiple distinct pneumococcal populations. Clusters of single nucleotide polymorphism (SNP) frequencies below 100% were indicative of the presence of multiple pneumococcal haplotypes. Sample H4IBM7 demonstrated two SNP clusters, one at 20% and the other at 80% which were designated as the minor and major strain, respectively. The reads from both strains were separated using a SNP frequency cut-off of 50%. The major strain from H4IBM7 was genetically more similar to the linked isolate H4IAM8 (distance 0.11 nuc sub/site) compared to the minor strain to H4IBM7 (distance 0.44 nuc sub/site) ( Figure 1).

### Putative transmission pairs identified with consensus SNP phylogenetic reconstruction

A maximum-likelihood (ML) phylogeny of the five putative transmission pairs was reconstructed from the consensus SNP sequences of the respective cross-sectional samples. The tree confirmed the clustering of isolate pairs that belonged to the same serotypes and were collected from the same households ( Figure 3A). The average genetic distance between the putative source-recipients pairs was 0.045 nuc sub/site (range, 0.038–0.057 nuc sub/site).

Consecutive-visit swabs from the same individuals were also included in the consensus SNP tree reconstruction for households three and eight. The phylogeny revealed there was an insufficient phylogenetic signal to distinguish samples collected from the same individual a month apart compared to samples collected cross-sectionally from transmission pairs within a month after the transmission event ( Figure 3B).

**Figure 4.**
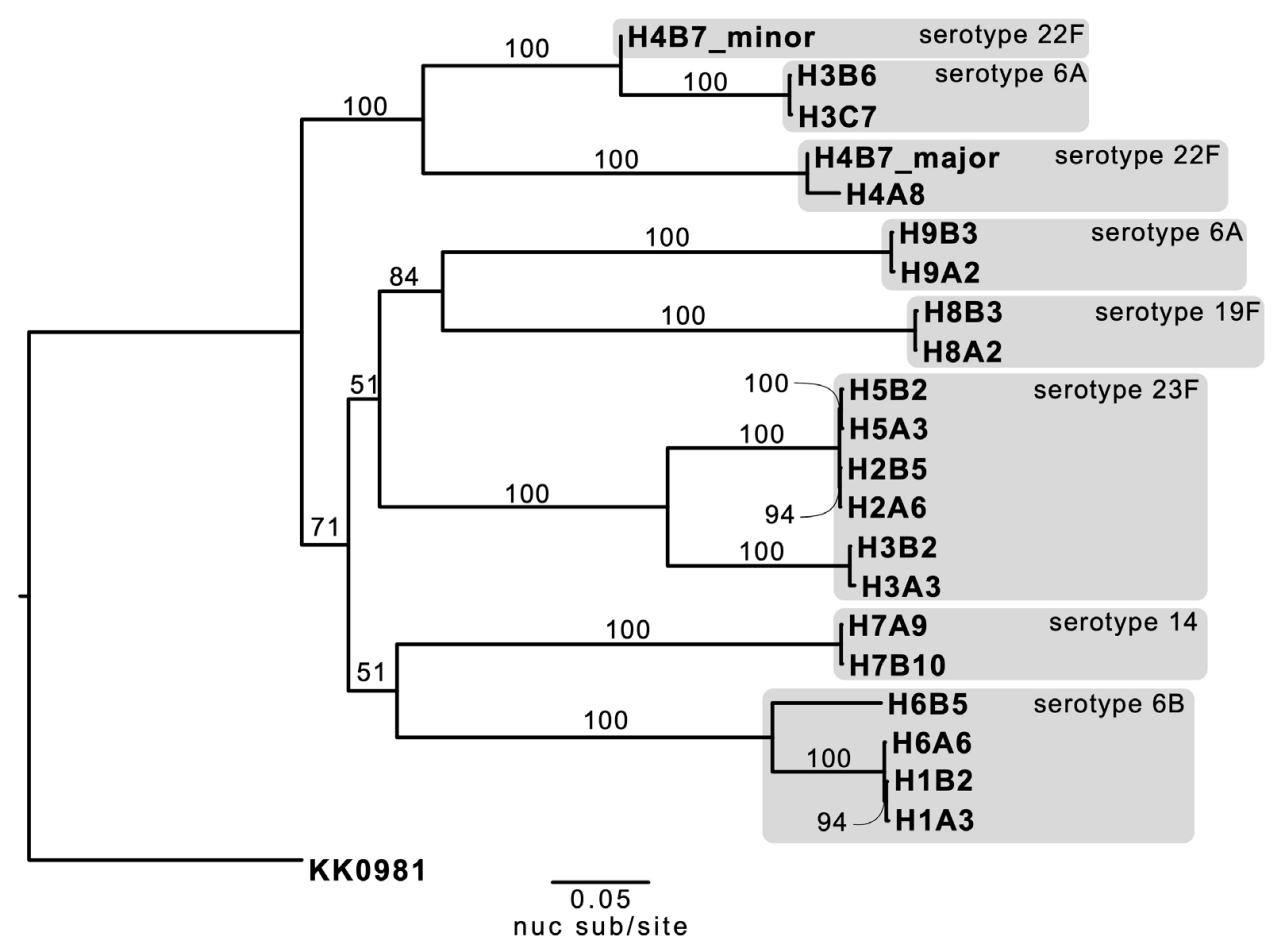
Maximum-likelihood phylogeny of the 20 *S. pneumoniae* genomes from the 10 pairs of isolates from subsequent visits rooted to the reference genome, KK0981. The consensus SNP tree was reconstructed from an alignment of all polymorphic sites along the genomes (51,682 bp). Branch supports ≥50%, as determined by 1,000 bootstrap replicates, are denoted on the relevant branches. Branch length represents nucleotide substitutions per site (nuc sub/site), as denoted by the scaled bar. Within-serotype clustering is highlighted in grey boxes.

In the sensitivity analysis, we reconstructed a tree using consensus SNP sequences from likely transmission pairs but taken at subsequent visits e.g. one month apart (N=10 pairs). Of the 10 putative pairs, nine pairs (90%) clustered concordantly with the epidemiological data with ≥90% bootstrap support. Amongst those clustered pairs, eight demonstrated short genetic distances (≤0.10 nuc sub/site) except for pair H4IBM7_major and H4IAM8, which could be due to the imperfect haplotype reconstruction, and for H6IAM6 and H6IBM5 we found >0.10 nuc sub/site difference between the two isolates suggesting a potential indirect transmission event ( Figure 4).

### Direction of transmission using within-host genomic variation

The direction of transmission was inferred from the five pairs of same-visit samples using Phyloscanner, a tool that implements a sliding window approach across the genomes and reconstructs sub-trees using the reads present in a given window. For each sub-trees reconstructed, the source of the infection is determined through a modified maximum-parsimony ancestral state reconstruction inference, where the most likely identity of the pair member is inferred at each node.

**Figure 5.**
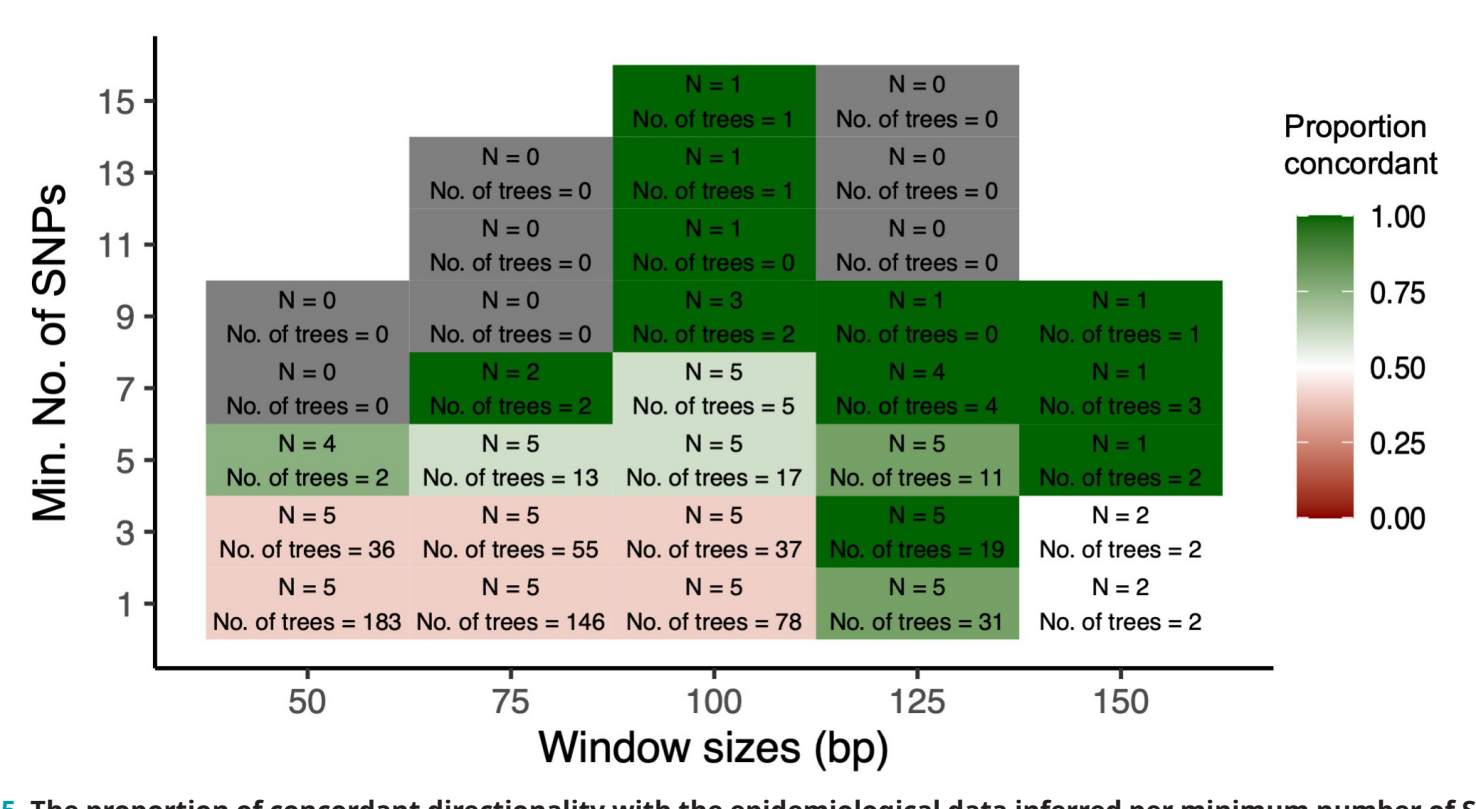
The proportion of concordant directionality with the epidemiological data inferred per minimum number of SNP per read (1,3,5,7,9,11,13, or 15 SNP) and read window sizes (50, 75, 100 125, or 150 bp). Inference from samples collected during the same visit. Green and red-coloured boxes denote the proportion of pairs for which the inferred transmission direction was concordant with the epidemiological data, green is equivalent to 100% and red is equivalent to 0%. White boxes denote equal distributions of concordant and discordant inferred directions (proportion = 0.50). While grey boxes denote that phylogenies were generated, however, they were classified as “unlinked” or “ambiguous directions” and empty boxes denote that no sub-trees were generated for this combination of window size and SNP. The “N” represents the number of pairs analysed for the respective window size and SNP combination and the “N of Trees” is the average number of sub-trees used for the direction of transmission for those pairs analysed.

**Figure 6.**
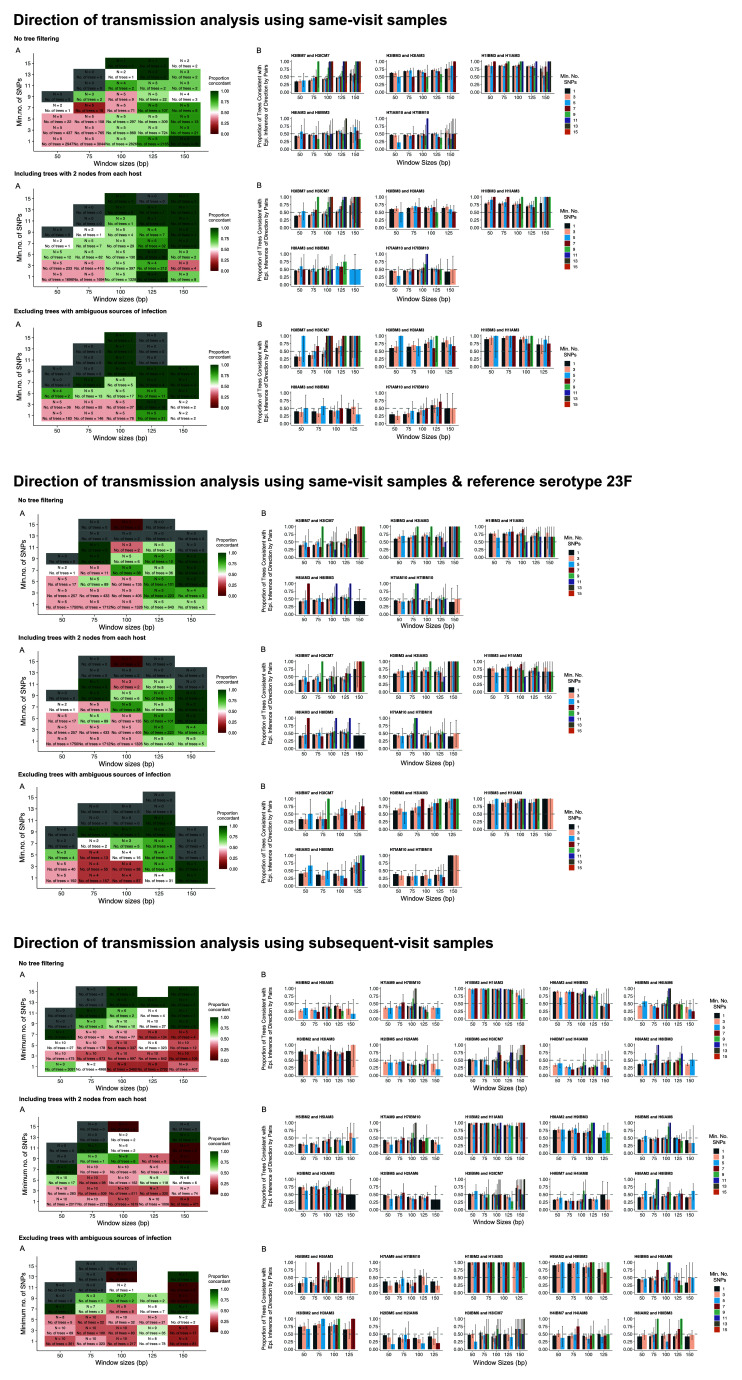
Sensitivity analysis inferring the direction of transmission. The proportion of concordant inferred directionality per minimum number of SNP per read (1,3,5,7,9,11,13, or 15) and read window sizes (50, 75, 100 125, or 150 base pairs). ( **A**) Inference from samples collected during the same visit. Green and red-coloured boxes denote the proportion of pairs for which the inferred direction of transmission was concordant with the epidemiological data, green is equivalent to 100% and red is equivalent to 0%. White boxes denote equal distributions of concordant and discordant inferred directions (proportion = 0.50). While grey boxes denote that phylogenies were generated, however, they were classified as “unlinked” or “ambiguous directions” and empty boxes denote that no sub-trees were generated for this combination of window size and SNP. The “N” represents the number of pairs analysed for the respective window size and SNP combination and the “N of Trees” is the average number of sub-trees used for the direction of transmission for those pairs analysed. ( **B**) The proportion of sub-trees concordant with the epidemiological data, for each pair, with the different combinations of window sizes and minimum number of SNP represented by the coloured bars.

We conducted a total of 200 inferences of the direction of transmission conducted across the five transmission pairs. The inferences were generated from a combination of varying sliding window sizes (50, 75, 100, 125, 150 bp) and varying minimum number of SNP (1,3,5,7,9,11,13,15 SNP) in the sub-tree reconstruction as these parameters would most likely affect the phylogenetic signal. Sub-trees were filtered for a minimum of two reads per individual and a clear ancestral state assignment to one of the individuals. This resulted in 102 inferences (51.5%) being viable to infer the direction of transmission. As expected, increasing either the minimum number of SNP threshold or the window size decreased the number of sub-trees included in the inference ( Figure 5 and Figure 6).

For small window size and a low SNP threshold, concordance with the epidemiologically inferred direction of transmission was two to three out of the five pairs, with 50% being the expected concordance if inference was no better than random chance. The proportion of pairs in which the direction of transmission was inferred in concordance with the epidemiological records generally increased with larger window sizes and/or more SNP. At least four out of the five inferred directions of transmission were concordant if using sliding window sizes of 125 bp, however, no analyses with further increased window size were possible due to the lack of samples with sufficient read lengths in the present sequencing approach ( Figure 5).

**Figure 7.**
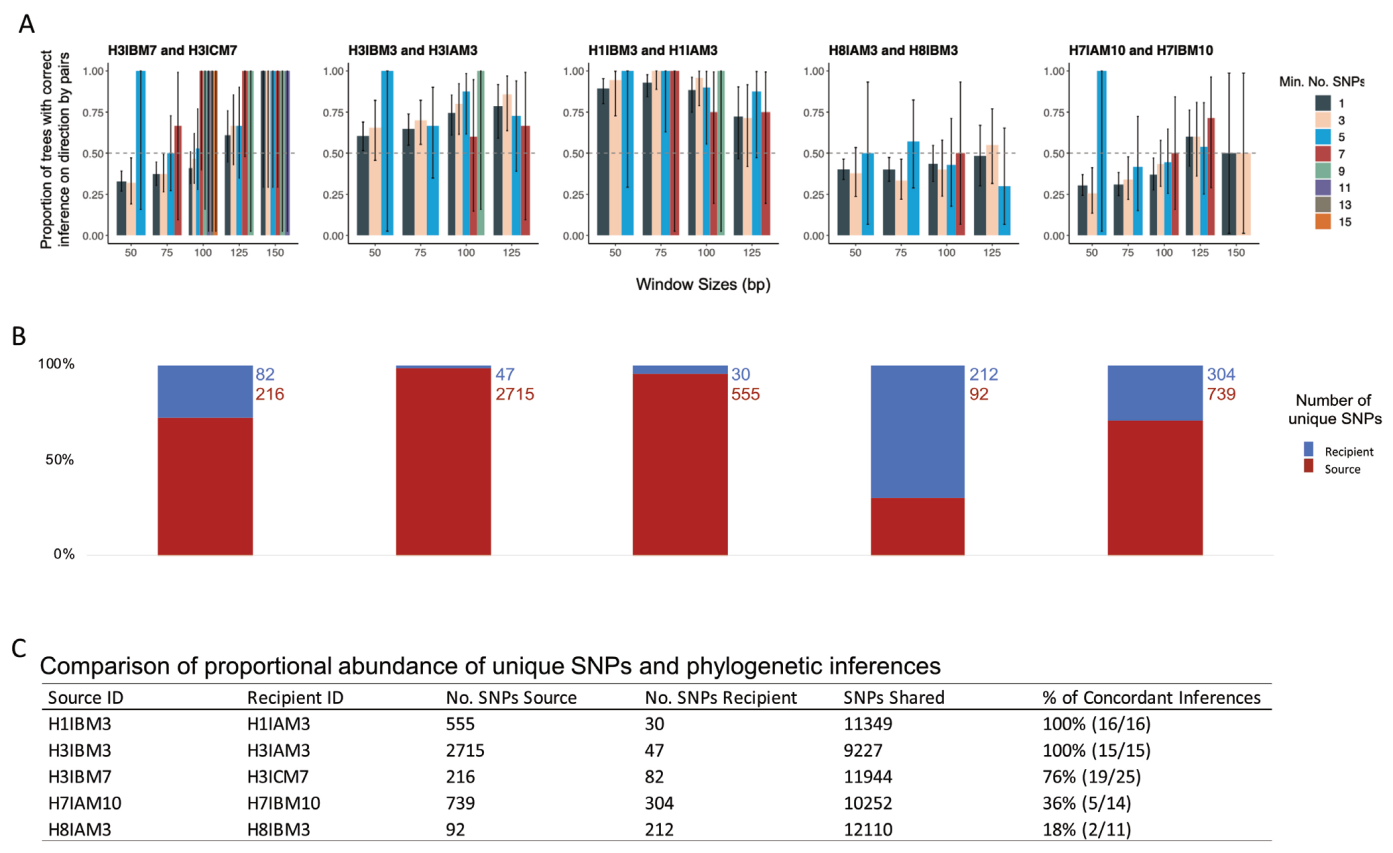
( **A**) The proportion of sub-trees concordant with the epidemiological data, for each pair, with the different combinations of window sizes and minimum number of single nucleotide polymorphism (SNP) represented by the coloured bars. ( **B**) Proportional abundances of unique SNP in source-recipient pairs. The proportional abundances are observed in source and recipients with the red bar denoting the percentage of unique SNP from the suspected source of infection, while the blue bar is the recipient ( **C**) The raw number of unique SNP detected for the source, recipient, and variants that are shared. The % of concordant inferences represents the number of inferences (combinations of a minimum number of SNP and window sizes) that were analysed and concordant with the epidemiological data.

Increasing the sliding window size and/or minimum number of SNP resulted in a higher level of concordance with the epidemiological evidence in the directionality inferred for pairs H3IBM7 and H3ICM7; and H7IAM10 and H7IBM10. Pairs H3IBM3 and H3IAM3; and H1IBM3 and H1IAM3 demonstrated consistently concordant directionality independent of window size and/or minimum number of SNP. Conversely, the pair H8IAM3 and H8IBM3 demonstrated consistent discordant directionality ( Figure 7A).

In a sensitivity analysis using a different reference genome, serotype 23F, the findings were qualitatively similar in the direction of transmission analysis with subsequent-visit sample pairs, albeit the association was less apparent ( Figure 6).

### Within-host diversities of source-recipient pairs

The proportion of unique SNP in the source-recipient pairs when sampled during the same visit was used as a proxy for the presence of a transmission bottleneck effect; expecting the source to have had more time to evolve before transmitting a subset of the acquired within-host heterogeneity and thus presenting more unique SNP than the recipient.

The average number of polymorphic sites between the source and recipient of a pair was 11,975 per transmission pair (SD, ± 1067). The source and recipient of all five same-visit pairs shared a large proportion of SNP (mean 91.6%; SD, ± 8.6%). The source of infection as determined by the epidemiological records had a higher proportion of unique polymorphic sites compared to the recipient for four of the five pairs; 7.3% vs 1.1% (range of unique SNP source vs recipient, 0.7%–22.6% vs 0.3%–2.7%). The only pair where the putative sources had a smaller proportion of unique polymorphic sites was H8IAM3 (source) and H8IBM3 (recipient); the pair was found to consistently suggest a direction of transmission discordant to the epidemiological records ( Figure 7B, Figure 3C).

The direction of transmission inferred by the larger number of unique SNP was compared to that inferred by Phyloscanner. Pair H3IBM3 and H3IAM3 had the largest difference in the proportion of unique SNP as previously mentioned, while pair H1IBM3 (source) and H1IAM3 (recipient) had a relatively moderate difference with 4.7% and 0.03% unique SNP, respectively. Both of these pairs had a consistent concordant transmission direction across all permutations of window sizes and minimum number of SNP. Conversely, pair H3IBM7 (source) and H3ICM7 (recipient) had the smallest differences in the proportion of unique SNP and mixed inferences. Further, pair H7IAM10 (source) and H7IBM10 (recipient) had relatively large differences in the proportion of unique SNP and also had mixed inferences ( Figure 7B). Interestingly, the only pair that exhibited a larger proportion of unique SNP in the recipient compared to the source, H8IAM3 (source) and H8IBM3 (recipient), had a consistent discordant directionality despite an increase in window sizes or minimum number of SNP ( Figure 7).

### Estimation of the within-host rate of nucleotide substitution

**Figure 8.**
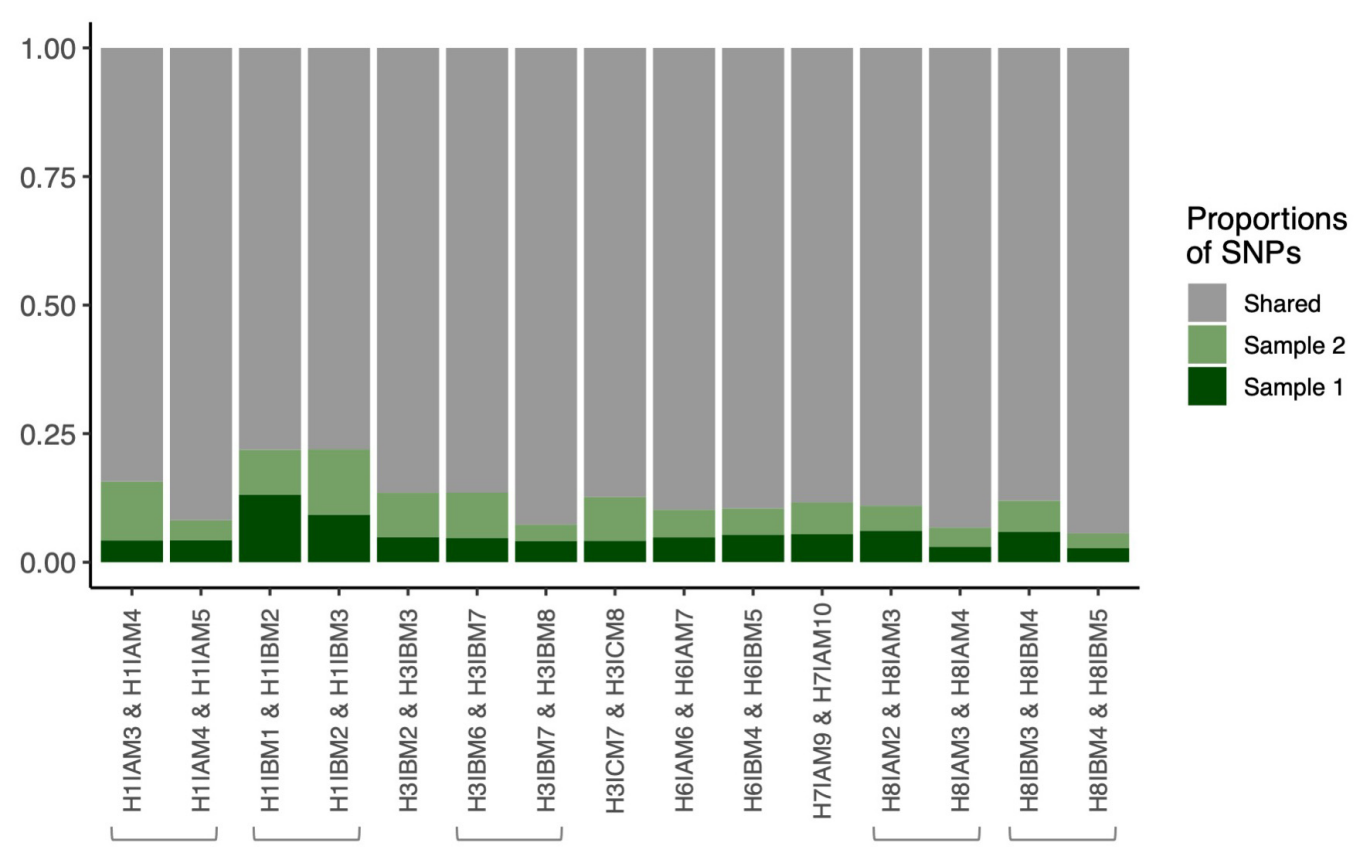
Proportional abundances of unique single nucleotide polymorphism (SNP) count from 1-month intervals from within-host longitudinal samples. Where individuals had at least two consecutive swabs, the first time point was compared to the second time point and subsequently, the second time point was compared to the third time point. Instances of individuals having more than two consecutive swabs are denoted by the grey brackets. The light green represents the proportion of SNP from the first time point and the dark green represents the count from the second time point of the consecutive sets. The grey represents the shared SNP counts present in both time points. The proportions of the unique number of SNP are explicitly written within each of the corresponding coloured bars.

The within-host rate of nucleotide substitution for *S. pneumoniae* was 65 SNP/month (range, 15–1539 SNP) and the within-host evolutionary rate 1.8E-5 nucleotide substitutions/site/year (range, 6.0E–5, 1.7E-6) ( Figure 8).

## Discussion

In this study, a genomic approach was used to infer the direction of *S. pneumoniae* transmission and cross-validated with the direction of transmission inferred from epidemiological evidence. We found that linkage was concordantly identified from reconstructed phylogenies in all five of the same-visit pairs and nine of the 10 subsequent-visit pairs. Albeit, the phylogenetic linkage of the same-visit pairs may in part be attributable to the serotype heterogeneity. To address this, paired isolates from subsequent months were assessed where there is more serotype homogeneity and more transmission pairs and the phylogenetic reconstruction revealed distinguishable linkage in addition to the indistinguishable linkage of pairs within their respective serotypes. The indistinguishable linked pairs within a serotype cluster could be due to the difference in sampling time between the two consecutive months which could contribute to genetic drift and the accumulation of variation in the recipient of the infection. These results imply that linked pneumococcal infection is identifiable from genomic data alone, however, more stringent phylogenetic criteria e.g. more conservative bootstrap cutoff or larger intra-cluster genetic distance thresholds might have to be placed in settings where there is more serotype homogeneity and less population diversity.

The two parameters that were likely to affect the probability of identifying the concordant source-recipient relationship within a transmission pair using a sliding-window phylogenetic approach were the sliding window sizes and the minimum number of SNP present within those windows. These two parameters indeed impact the phylogenetic signal of the read alignment used to reconstruct the sub-trees; e.g. on the capacity to reconstruct a robust phylogeny from which conclusions can be drawn with sufficient statistical certainty. Under optimal conditions, the direction of transmission was concordant between the epidemiological records and phylogenetic inference for all five same-visit transmission pairs with a window size of 125 bp and a minimum number of three SNP. Moreover, based solely on the genomic data, the phylogenetic inference and the transmission bottleneck analysis were concordant in all five same-visit pairs.

In general, these results suggest an increased concordant direction inferred from combinations of longer window sizes and a larger minimum number of SNP, however, the sample size and the maximum window size were too low to allow a definitive conclusion. Hence further studies are needed to determine whether higher coverage and/or read lengths can increase the phylogenetic signal for inferring the direction of transmission. More sequencing coverage would increase the phylogenetic genetic signal by detecting minor variations between source-recipient pairs while the longer reads would aid in the genome assembly and thus provide more robust genomes.

To our knowledge, the only studies that have attempted to validate genomic approaches against epidemiological data on the direction of transmission were using HIV transmission pairs ( Rose *et al.*, 2020; Villabona-Arenas *et al.*, 2022; Zhang *et al.*, 2021). Villabona-Arenas *et al.* investigated the phylogenetic inference of known transmission direction of HIV-1 transmission partners. They observed an increase in correct transmission direction up to 93% when inferring from paraphyletic-monophyletic tree topology highlighting the importance of sufficient intra-host diversity to distinguish HIV-1 populations amongst partners ( Villabona-Arenas *et al.*, 2022). Rose *et al*. looked at HIV transmission partners where the accuracy of transmission direction was inferred concordantly for 55%–74% of the pairs and the range was dependent on the sequencing and inference methods used ( Rose *et al.*, 2020). While a more recent study from Zhang *et al*., using the same cohort as Rose *et al.*, increased the accuracy up to 93.3% ( Zhang *et al.*, 2021). Zhang *et al.* speculated the higher accuracy for inferring transmission direction compared could be attributable to higher sequencing coverage in addition to the longer sequencing reads up to 400 bp. Zhang *et al.* also used Phyloscanner for their analysis and similarly explored the impact of varying window sizes across the entire HIV genome. They reconstructed sub-trees between 280–400 bp in 20 bp increments and observed higher accuracy using larger window sizes. This prompts further investigation to assess if increased coverage and/or sequencing reads would also increase phylogenetic signal in bacterial pathogen transmission.

The evolutionary rate of bacteria is relatively slow compared to fast-evolving RNA viruses such as HIV where bacteria evolve between 10 ^-7^ to 10 ^-5 ^substitutions/site/year and amongst the fastest evolving pathogens, between 10 ^-4^ to 10 ^-3 ^substitutions/site/year ( Didelot *et al.*, 2016). The relatively slower evolutionary rate of bacteria to viruses substantially affects the number of accumulated mutations, therefore, the number of genetic fingerprints to link transmission pairs and its direction.

The comparison of within-host bacterial diversity within the transmission pairs showed evidence of a transmission bottleneck of varying strengths, with a higher percentage of unique SNP in the source’s bacterial population compared to the recipient’s in four of five of the studied pairs implying the direction of transmission according to the epidemiological records could be incorrect which could be explained by false negative sampling ( Thindwa *et al.*, 2021). This directed reduction of diversity could aid in determining the direction of transmission when the latter is not known.

Hall *et al.* used a similar approach to investigate the transmission direction of Methicillin-resistant *Staphylococcus aureus* (MRSA), in a high-transmission setting ( Hall *et al.*, 2019). They observed varying transmission bottleneck strengths among their source-recipient pairs. The bottleneck strength ranged from strong where a single lineage was transmitted from the source to the recipient to weak where the transmission pairs shared multiple lineages, however, the direction was ambiguous. In conjunction with our study, this suggests the presence of a transmission bottleneck for bacteria, however, the strength of the bottlenecks is not associated with a higher probability of inferring the concordant direction of transmission. In other words, while we observed more unique SNP in the source of the infection compared to the recipient, a larger proportion of unique SNP in the source compared to the recipient is not associated with higher chances of inferring the concordant direction. These results imply that the observed bottleneck effect is not random and a comparison of the number of unique SNP in the members of a suspected transmission pair can aid in supporting the direction of transmission inferences, under the assumption that the recipient will be the individuals with the bacterial population exhibiting the least number of unique SNP.

The inclusion of additional longitudinal samples from the same individual, sampled over a couple of months, confounded the ability to detect true transmission pairs. This suggests that there is relatively little within-host diversity within that time frame to distinguish transmission pairs from within-host samples. The evolutionary rate that was extrapolated from the SNP accumulated over time is relatively small and there would be less diversity accumulated especially when looking at a 1-month or even 2-month sampling time difference. The within-host evolutionary rate for *S. pneumoniae* that we estimated is similar to the estimates by Chaguza *et al*. who looked at the natural colonisation of longitudinal samples with estimates around 10 ^-5 ^substitutions/site/year for most serotypes and as low as 10 ^-6^ substitutions/site/year for serotype 19A ( Chaguza *et al.*, 2020). Moreover, the rates are dependent upon the carrier, serotype, and colonisation episodes, suggesting the importance of the host-microbe interaction during the evolution of pneumococcus.

Rather than longitudinal within-host diversity, Hall *et al*. looked at within-host MRSA diversity between samples from different body sites and similarly saw no evidence for decreased or increased genetic diversity between the within-host samples. Other studies, in the context of *Clostridioides difficile* and slow-evolving bacteria such as *Mycobacterium tuberculos*is, observed difficulty capturing within-host level diversity from whole-genome sequences ( Balaji *et al.*, 2019; Martin *et al.*, 2018). As expected, the within-host diversity of bacteria is difficult to capture, especially in the absence of relatively high coverage sequencing data. While most pneumococcal infections are dominated by a major serotype, there are settings of mixed high carriage rates, and being able to capture the within-host diversity is crucial for understanding transmission dynamics ( Kamng’ona *et al.*, 2015).

The transmission directions that were phylogenetically inferred and discordant with the epidemiological records could be attributable to multiple factors and inherent limitations of the studies. The first is the imperfect sensitivity of the swab collection in combination with the imperfect sensitivity of the culturing technique to detect pneumococci and identify the dominant serotype. Pneumococcal testing has been previously reported with 85% sensitivity (95% CI, 73%–94%) which would result in up to 15% false-negative tests ( Abdullahi *et al.*, 2007; Thindwa *et al.*, 2021). With false-negative testing, a carriage episode could have been missed and thus led to a different interpretation of transmission direction based on the epidemiological data on the sequence of pneumococcal positivity within the households.

The second includes potential unsampled intermediary transmission partners that were not included in the study. Since the transmission is predominantly through close contact and within households, it is unlikely an individual outside of the household is introduced to the transmission chain. However, the possibility of an unsampled person within the link cannot be discarded. If there was an intermediary individual within the chain between the time of sampling of the source and recipient pairs, then the directionality would be more difficult to determine due to the decreased mutation similarities between the source and recipient.

The third factor includes the phylogenetic uncertainty that is limited by the short-read fragments. An increase in read lengths would result in improved genome assembly and therefore increased genomic signal ( Mantere *et al.*, 2019). Other sequencing methods such as PacBio can yield longer read lengths, up to 10 kbp, and should be further investigated and assessed if improved genome assemblies improve phylogenetic inference in assessing the directionality of transmission.

In summary, in this pilot study we find evidence that conventional NGS may offer too little phylogenetic signal to allow robust inference for the direction of transmission for cross-sectionally sampled pairs of pneumococcal carriage, but that with increased sequencing depth and particular fragment size, such inference may be possible. This motivates further studies to explore the feasibility and limits of inference of who infected whom with pneumococci from genomic data.

## Data Availability

The whole-genome sequencing data has been made available for download on the European Nucleotide Archive under study accession “PRJEB60532” including the corresponding sample alias that are referenced in this manuscript. Bioproject: Phylogenetic inference of pneumococcal transmission from cross-sectional data, a pilot study. Accession number: PRJEB60532. https://identifiers.org/bioproject:PRJEB60532. Nucleotide: Streptococcus pneumoniae DNA, complete genome, strain: KK0981. Accession number: AP017971. https://identifiers.org/nucleotide:AP017971 ( Chiba *et al.*, 2017). Nucleotide: Streptococcus pneumoniae ATCC 700669, complete sequence. Accession number: NC_011900. https://identifiers.org/nucleotide:NC_011900 ( Croucher *et al.*, 2009).
