# Supplementary material for: Phylogenetic inference of pneumococcal transmission from cross-sectional data, a pilot study
Source: Wellcome Open Res. 2023 Oct 6;8:427. [Version 1] doi: 10.12688/wellcomeopenres.19219.1 (PMC11024593; doi:10.12688/wellcomeopenres.19219.1)
Supplement: Peer review history

# Reviewer report: Reviewer response for version 1

**DOI:** 10.21956/wellcomeopenres.21301.r77547

**Published:** 2024-04-17

**Authors:** Leah J Ricketson

**Affiliations:** 1 University of Calgary, Calgary, Canada

This is an interesting study using genomics to understand transmission dynamics.

 I feel you could make more of a case in the introduction and discussion for the importance of why we need to understand the direction of transmission. Does knowing the direction of transmission provide benefit to the management of disease and have clinical relevance?

 Research is now showing carriage is best identified through PCR as much is missed by culture. Did you or could you conduct PCR detection of S. pneumo for the samples rather than culture only? You do address this limitation in your discussion, which is good.

 There is also some evidence from carriage studies suggesting adults more often carry bacteria in the oropharynx so carriage studies may need to sample differently for adults to capture carriage adequately. It appears in the Hussein study that only NP swabs were collected.

 You mention that a individual outside the household being unlikely involved in the transmission chain, but with kids under 3 if they are in daycare or other exposure to young children then that might not be unlikely as children are excellent at sharing saliva and sputum at that age. Did you have any information on daycare status for the children?

 I would be interested to know whether the direction of transmission is child to adult or adult to child or between children in your study. Is this something that could be provided? Children are often thought of as the reservoir and the ones spreading S. pneumoniae.

Is the work clearly and accurately presented and does it cite the current literature?

Yes

If applicable, is the statistical analysis and its interpretation appropriate?

Yes

Are all the source data underlying the results available to ensure full reproducibility?

Yes

Is the study design appropriate and is the work technically sound?

Yes

Are the conclusions drawn adequately supported by the results?

Yes

Are sufficient details of methods and analysis provided to allow replication by others?

Yes

Reviewer Expertise:

Epidemiology of S. pneumoniae: observational research looking at disease and serotype trends over time with the influence of vaccines, vaccine schedule changes and other factors influencing disease trends.

I confirm that I have read this submission and believe that I have an appropriate level of expertise to confirm that it is of an acceptable scientific standard.

# Reviewer report: Reviewer response for version 1

**DOI:** 10.21956/wellcomeopenres.21301.r77550

**Published:** 2024-04-15

**Authors:** Matthew A. Croxen

**Affiliations:** 1 University of Alberta, Edmonton, Alberta, Canada

Hackman et al have attempted to recapitulate the direction of pneumococcal transmission with households. This is of great interest not only for researchers, but more broadly for public health and infection control practitioners where it could be hugely beneficial to reconstruct transmission networks from genomics data, especially in the absence of good epidemiological support. Although a pilot, I think this work should be of interest to many in the field. I hope my comments below will be helpful in improving the clarity of the manuscript. I will also refrain from repeating a few similar comments that I had that the other reviewer discussed, especially around the choice of references where I agree a "within household reference" should have been used for each pairing for more resolution based on more genome coverage, and fewer variant sites relative to the reference.

- In the Genomic Serotyping section, you describe predicting the serotypes, but then proceed to describe a mapping (bwa) and SNP calling (Freebayes). Calling SNPs this way technically isn't "serotyping", so may be require it's own section. Second, I feel like this is how you are doing the haplotype reconstruction - but it really isn't described that way, so I would suggest more information on how haplotypes were reconstructed. My guess is that you are assuming that the minor alternate allele frequency is consistent within the sample across the genome.
- I also had a few questions about the microbiological work. It seems that it would have been more ideal to start from a single colony for a better shot at homogeneous growth. A sweep from a plate has the possibility of adding more genetic heterogeneity.
- I would also suggest a little clarification on the sensitivity studies, where you switched from using a serotype 3 reference strain to a serotype 23F. In the results section when you discussed "sensitivity studies", it wasn't always clear which reference was being used.
- I quite like the exemplars in Figure 1 and Figure 2. It makes this part of the setup clear for the reader.
- What is meant by "read windows"? Are you talking specifically about the individual mapped reads? Similarly, with the SNP per read, would you expect to have 15 SNPs in a single read? I guess its possible depending how diverse your reference strain is, which goes back to the comment above about within-household references, and also noted by the first reviewer.
- Could you perhaps comment more on the within-host nucleotide substitutions? 65 SNPs/month seemed like a lot to me; 1539 seems really high.
- Finally, I would suggest that you stay consistent (in the context of this manuscript) with the use of colonization vs infection. My understanding was that the original collection was due to colonization/carriage, and not infection.

Very interesting work, I look forward to future developments.

Is the work clearly and accurately presented and does it cite the current literature?

Yes

If applicable, is the statistical analysis and its interpretation appropriate?

Yes

Are all the source data underlying the results available to ensure full reproducibility?

Yes

Is the study design appropriate and is the work technically sound?

Yes

Are the conclusions drawn adequately supported by the results?

Yes

Are sufficient details of methods and analysis provided to allow replication by others?

Partly

Reviewer Expertise:

Infectious disease genomics, public health genomics

I confirm that I have read this submission and believe that I have an appropriate level of expertise to confirm that it is of an acceptable scientific standard, however I have significant reservations, as outlined above.

# Reviewer report: Reviewer response for version 1

**DOI:** 10.21956/wellcomeopenres.21301.r75056

**Published:** 2024-04-01

**Authors:** Taj Azarian

**Affiliations:** 1 Burnett School of Biomedical Sciences, University of Central Florida, Orlando, Florida, USA

Thank you for the opportunity to review “Phylogenetic inference of pneumococcal transmission from cross-sectional data, a pilot study” by Hackman and colleagues. The authors sought to explore the directionality of pneumococcal transmission within household using sequencing data from cross-sectional samples. By analyzing within-host pneumococcal diversity using the bioinformatics tool Phyloscanner, they find concordance between epidemiologically and phylogenetically inferred transmission pairs. While I have several questions regarding the methods, I find their analysis of great interest to pneumococcologists and the bacterial genomics field at large. As the authors note, published examples of the application of phylogenetic tools to infer pneumococcal transmission is largely limited. Below I share more specific comments. While they appear extensive, they are not critical of the design of the study or interpretation of the results, and overall, I really enjoyed the paper.

 My most significant comment relates to the SPN culture, isolation, and sequencing process. First, in the “Isolate culturing and WGS” section, I was not clear what was done in the initial study vs the present study. This should be detailed further. In addition, they should clearly define the terms sample, population, strain, isolate, haplotype etc as it relates to their study. For example, they state, “SPN isolates obtained from the culture plates directly inoculated with the swab in the original study” and in another section “isolates were cultured and whole plate scrapes were processed for WGS.” To me, an isolate is a distinct colony that lacks any nucleotide diversity. A plate sweep of colonies growing on selective media inoculated directly using the collection swab or from culture enriched broth inoculated using the collection swab would contain the entire SPN population, which I believe is what the authors are using to identify the within-host SPN diversity. As the authors’ approach hinges on their ability to capture this diversity, the reader should clearly understand how this was accomplished.  I believe that this component of the methods is so important that it warrants an infographic.  At present, this was difficult to ascertain. Similarly in the section regarding “multi-carriage detection”, there should be an explanation of the definition. Are these two separate serotypes/MLST/GPSCs or just highly diverged subpopulations of the same strain?

 Regarding the reference-based assembly approach, I was left wondering about what the impact of reference selection on the results, even considering the sensitivity analysis with the 23F strain. My approach would have been to use the de novo assemblies from each intrahost sample to identify the closest published reference genome and then use that for the reference-based assembly. I believe this would have gained more resolution in portions of the genome that were present in the intrahost sample but not in the reference. I think this may be worth exploring or at least including a justification of the current approach. Also, can the authors clarify if the “SPN read match %” in Table 2 is the proportion of reads mapped to the reference?  If so, then mid-to-low 80s may justify using a closure matching reference for each population as a closure reference would have likely yielded high 90s. In Table 2, the authors should include reference genome coverage (i.e., proportion of unmapped portions of the genome). Last, is there any indication on how recombination impacted the analysis? Did the SNP criteria filtering account for SNPs introduced through recombination?

 The authors should detail how Phyloscanner works in more detail in the “Phylogenetic reconstruction of putative transmission pairs” section, specifically stating that the input is a BAM file that theoretically includes reads from multiple within-host bacterial isolates. I read the original Phyloscanner paper when it was published, and I had to go back to confirm this; therefore, I feel it is important to include in the present paper.

 In Table 1, the authors should include the number of individuals in each household.  They currently include a nice discussion on the likelihood of unsampled transmission pairs, detailing the false negative rates of SPN identification. If information on the number of individuals in the households were included, the reader could make some inference about the likelihood of missing transmission links.

 Similar to the comment above, the authors don’t mention the likelihood of backward transmission following the initial transmission event. I don’t know if this has ever been documented for SPN, but it seems possible. This would certainly confound the phylogenetic inference method used in the study.

 The authors state that increased sequence length would have improved their ability to infer the directionality of transmission. Indeed, I wished they had used a V3 600 cycle kit (but I certainly understand how these things occur). Is there any thought on the magnitude of improvement that could be gained by using 300 bp reads or even 3000+ bp reads from and ONT platform?

 Minor Comments

- I suggest changing the red and green colors on the figure to something colorblind friendly. Even black/greyscale would be suitable.
- The authors state that Kraken was used for QC to remove non-SPN reads. They should include how many samples had non-SPN reads removed as well as the proportion of those reads.
- In the results, it states that the average number of polymorphic sites between the source and recipient of a pair was 11,975 per transmission pair.  Can the authors clarify whether this included the distance to the reference genome or if the reference genome sites were excluded?  This number just doesn’t sound correct, or I am not interpreting the result correctly. If it includes the reference genome, then the polymorphic sites only found in the reference should be removed.
- Was there any evidence of genome content differences among transmission pairs?

Is the work clearly and accurately presented and does it cite the current literature?

Yes

If applicable, is the statistical analysis and its interpretation appropriate?

Yes

Are all the source data underlying the results available to ensure full reproducibility?

Yes

Is the study design appropriate and is the work technically sound?

Yes

Are the conclusions drawn adequately supported by the results?

Yes

Are sufficient details of methods and analysis provided to allow replication by others?

Partly

Reviewer Expertise:

Bacterial genomics, phylogenetics, infectious disease epidemiology, Streptococcus pneumoniae, Staphylococcus aureus

I confirm that I have read this submission and believe that I have an appropriate level of expertise to confirm that it is of an acceptable scientific standard, however I have significant reservations, as outlined above.
